# Supplementary material for: Redox Responsive Copolyoxalate Smart Polymers for Inflammation and Other Aging-Associated Diseases
Source: Int J Mol Sci. 2021 May 25;22(11):5607. doi: 10.3390/ijms22115607 (PMC8198274; doi:10.3390/ijms22115607)
Supplement: Supplementary file 1 [file ijms-22-05607-s001.zip › ijms-1213616-supplementary.pdf]

Supplementary Materials

Redox Responsive Copolyoxalate Smart Polymers for Inflammation and Other Aging-Associated Diseases

Berwin Singh Swami Vetha [1], Angela Guma Adam [2] and Azeez Aileru [1,*]

[1] Department of Foundational Sciences and Research, School of Dental Medicine, East Carolina University, 1851 MacGregor Downs Road, MS 701, Greenville, NC 27834, USA; swamivethab20@ecu.edu

[2] Physio/Biochem/New Product Development Division, Cocoa Research Center Institute of Ghana, P.O. Box 8 Tafo, Ghana; angelaadamworld@gmail.com

* Correspondence: ailerua19@ecu.edu; Tel.: +252-737-7125

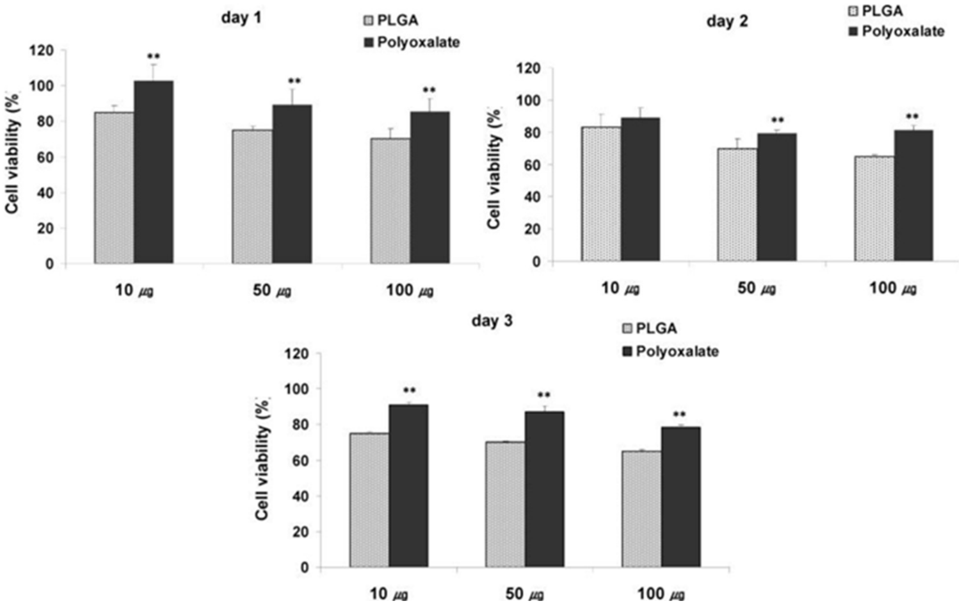

Figure S1. Cytotoxicity POx and PLGA nanoparticles in RAW 264.7 cells [Kim *et al.* 2010].

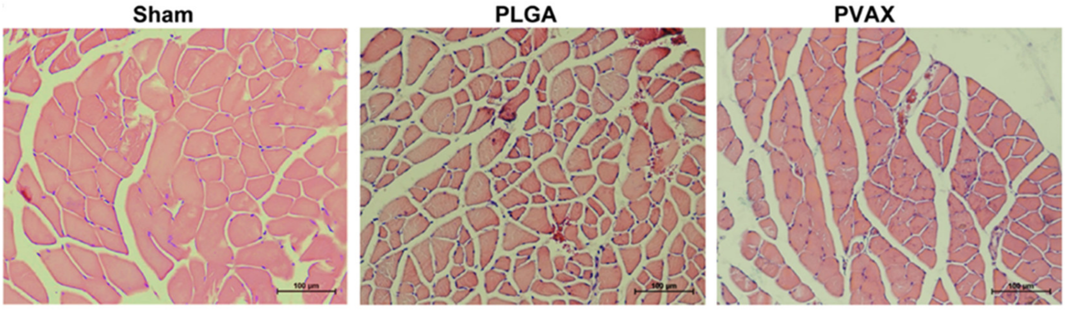

Figure S2. Tissue compatibility of CPOx (PVAX) and PLGA nanoparticles [Berwin *et al.* 2018].

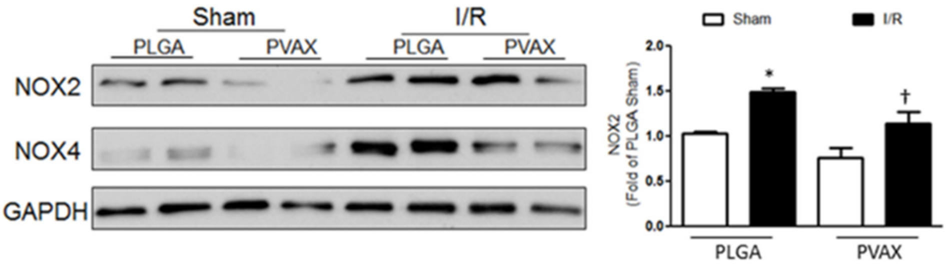

Figure S3. mRNA expression and quantification of of NADPH oxidase 2 of CPOx (PVAX) and PLGA [Bae *et al.* 2016].

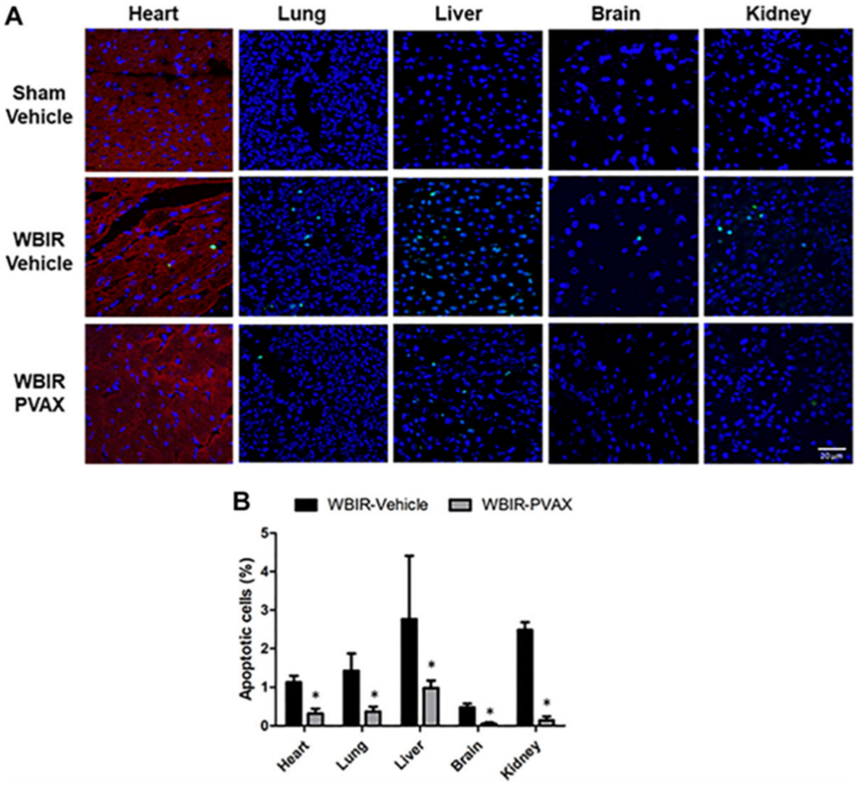

Figure S4. Effects of CPOx (PVAX) on apoptosis in multiple organs TUNEL fluorescent staining in heart, lung, liver, and brain, and kidney[4].

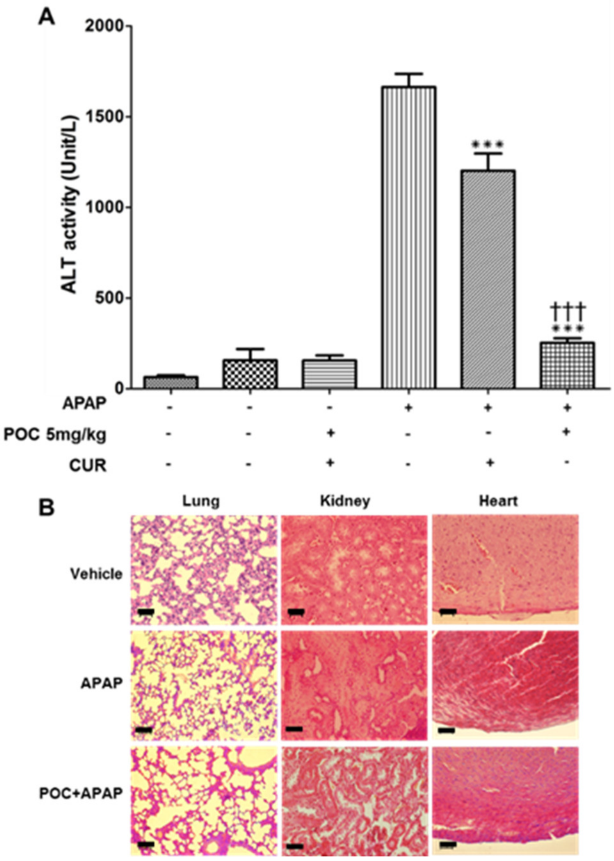

Figure S5. A) Serum ALT levels in APAP-intoxicated mice after POC particles treatment. Mean ± S.D. n = 3 *** P < 0.001 in relative to APAP treated group, ††† P < 0.001 in relative to APAP+ CUR treated group. **B**) Haematoxylin

and eosin staining of lung, kidney and heart treated with various formulation compared with vehicle treated group [Berwin *et al*. 2019].

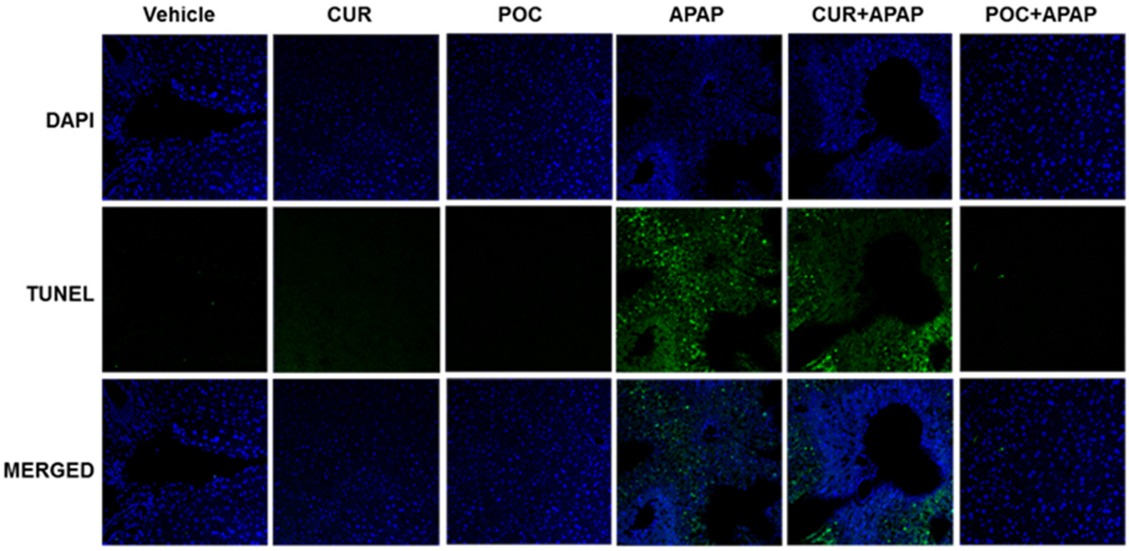

Figure S6. Liver tissues stained by TUNEL assay [Berwin *et al*. 2019]..

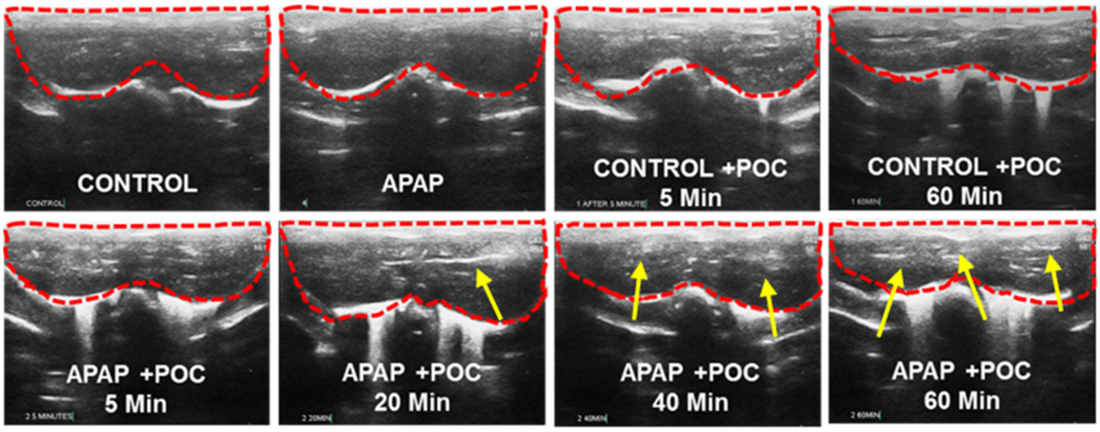

Figure S7. Ultrasound images of liver of APAP-intoxicated mice followed by POC nanoformulation treatment. Liver contour-dotted red lines and Echogenicity of POC particles- yellow arrows [Berwin *et al*. 2019].

Table S1. Biomedical application of Oxalate and copolyoxalate polymeric particles.

	Polymers	Molecule Incorporated	Biomedical Application	Reference
(i)	Polyoxalate	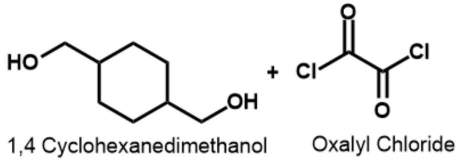	Chemiluminescence detection of ROS and drug loading	[1,6–8]
(ii)	Copolyoxalate			
	PAOX	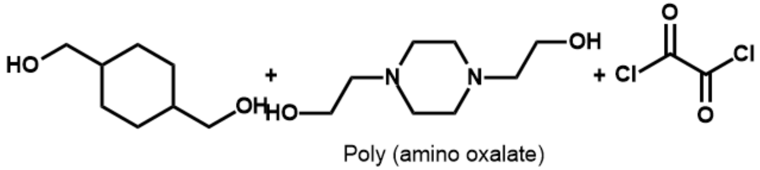	Encapsulation of aqueous insoluble drugs and Cytosolic drug delivery	[9]
	HPOX	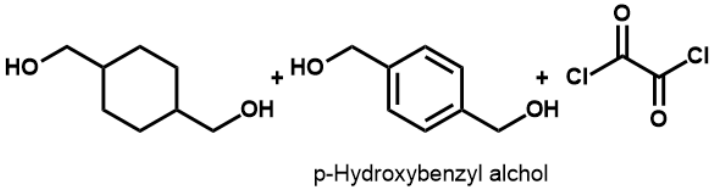	Inflammation, ischemia-reperfusion injury, chemi-dynamic therapy,	[10–13]
	PVAX	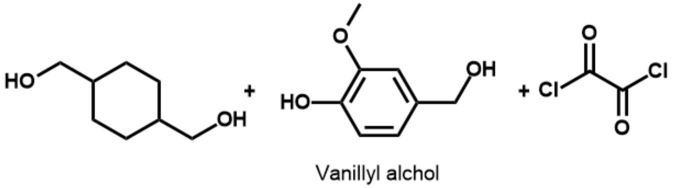	Myocardial infraction, muscle injury, liver injury, upper respiratory tract inflammation, wound healing, bioimaging.	[2,4,11–16]
	POC	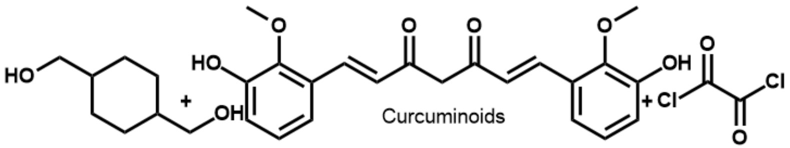	Liver injury and Bioimaging.	[5]

1. Kim, S.; Seong, K.; Kim, O.; Kim, S.; Seo, H.; Lee, M.; Khang, G.; Lee, D., Polyoxalate Nanoparticles as a Biodegradable and Biocompatible Drug Delivery Vehicle. *Biomacromolecules* **2010,** 11, (3), 555-560.
2. Berwin Singh, S. V.; Park, H.; Khang, G.; Lee, D., Hydrogen peroxide-responsive engineered polyoxalate nanoparticles for enhanced wound healing. *Macromolecular Research* **2018,** 26, (1), 40-47.
3. Bae, S.; Park, M.; Kang, C.; Dilmen, S.; Kang, T. H.; Kang, D. G.; Ke, Q.; Lee, S. U.; Lee, D.; Kang, P. M., Hydrogen Peroxide-Responsive Nanoparticle Reduces Myocardial Ischemia/Reperfusion Injury. *J Am Heart Assoc* **2016,** 5, (11), e003697.
4. Li, R.; Rhee, S. J.; Bae, S.; Su, S.; Kang, C. S.; Ke, Q.; Koo, Y. E.; Ryu, C.; Song, C. G.; Lee, D.; Kang, P. M., H2O2-Responsive Antioxidant Nanoparticle Attenuates Whole Body Ischemia/Reperfusion-Induced Multi-Organ Damages. *Journal of cardiovascular pharmacology and therapeutics* **2021,** 26, (3), 279-288.
5. Berwin Singh, S. V.; Jung, E.; Noh, J.; Yoo, D.; Kang, C.; Hyeon, H.; Kim, G.-W.; Khang, G.; Lee, D., Hydrogen peroxide-activatable polymeric prodrug of curcumin for ultrasound imaging and therapy of acute liver failure. *Nanomedicine: Nanotechnology, Biology and Medicine* **2019,** 16, 45-55.
6. Lee, D.; Khaja, S.; Velasquez-Castano, J. C.; Dasari, M.; Sun, C.; Petros, J.; Taylor, W. R.; Murthy, N., In vivo imaging of hydrogen peroxide with chemiluminescent nanoparticles. *Nature Materials* **2007,** 6, (10), 765-769.
7. Lee, E.; Kim, S.; Seong, K.; Park, H.; Seo, H.; Khang, G.; Lee, D., A biodegradable and biocompatible drug-delivery system based on polyoxalate microparticles. *Journal of biomaterials science. Polymer edition* **2011,** 22, (13), 1683-94.
8. Lee, C. J.; Kim, S.; Lee, H. G.; Yang, J.; Park, J.; Cha, S. R.; Lim, D.; Lee, D.; Khang, G., Preparation and Release Behavior of Atorvastatin Calcuim - Encapsulated Polyoxalate Microspheres. *Polymer-korea* **2014,** 38, 656-663.
9. Seong, K.; Seo, H.; Ahn, W.; Yoo, D.; Cho, S.; Khang, G.; Lee, D., Enhanced cytosolic drug delivery using fully biodegradable poly(amino oxalate) particles. *J Control Release* **2011,** 152, (2), 257-263.
10. Park, H.; Kim, S.; Kim, S.; Song, Y.; Seung, K.; Hong, D.; Khang, G.; Lee, D., Antioxidant and anti-inflammatory activities of hydroxybenzyl alcohol releasing biodegradable polyoxalate nanoparticles. *Biomacromolecules* **2010,** 11, (8), 2103-8.
11. Bae, S.; Park, M.; Kang, C.; Dilmen, S.; Kang, T. H.; Kang, D. G.; Ke, Q.; Lee, S. U.; Lee, D.; Kang, P. M., Hydrogen Peroxide‐Responsive Nanoparticle Reduces Myocardial Ischemia/Reperfusion Injury. *J Am Heart Assoc* **2016,** 5, (11), e003697.
12. Lee, D.; Bae, S.; Ke, Q.; Lee, J.; Song, B.; Karumanchi, S. A.; Khang, G.; Choi, H. S.; Kang, P. M., Hydrogen peroxide-responsive copolyoxalate nanoparticles for detection and therapy of ischemia-reperfusion injury. *J Control Release* **2013,** 172, (3), 1102-1110.
13. Eshun, D.; Saraf, R.; Bae, S.; Jeganathan, J.; Mahmood, F.; Dilmen, S.; Ke, Q.; Lee, D.; Kang, P. M.; Matyal, R., Neuropeptide Y3-36 incorporated into PVAX nanoparticle improves functional blood flow in a murine model of hind limb ischemia. *Journal of Applied Physiology* **2017,** 122, (6), 1388-1397.
14. Kim, G.-W.; Kang, C.; Oh, Y.-B.; Ko, M.-H.; Seo, J.-H.; Lee, D., Ultrasonographic Imaging and Anti-inflammatory Therapy of Muscle and Tendon Injuries Using Polymer Nanoparticles. *Theranostics* **2017,** 7, (9), 2463-2476.
15. Kwon, B.; Kang, C.; Kim, J.; Yoo, D.; Cho, B.-R.; Kang, P. M.; Lee, D., H2O2-responsive antioxidant polymeric nanoparticles as therapeutic agents for peripheral arterial disease. *International Journal of Pharmaceutics* **2016,** 511, (2), 1022-1032.
16. Jeong, D.; Kang, C.; Jung, E.; Yoo, D.; Wu, D.; Lee, D., Porous antioxidant polymer microparticles as therapeutic systems for the airway inflammatory diseases. *Journal of Controlled Release* **2016,** 233, 72-80.
